# Blunted hypothalamic ghrelin signaling reduces diet intake in rats fed a low‐protein diet in late pregnancy

**DOI:** 10.14814/phy2.12629

**Published:** 2015-12-10

**Authors:** Haijun Gao, Stephanie Sisley, Chandra Yallampalli

**Affiliations:** ^1^ Department of Obstetrics & GynecologyBaylor College of MedicineHoustonTexas; ^2^ Department of PediatricsBaylor College of MedicineHoustonTexas

**Keywords:** Diet intake, ghrelin signaling, hypothalamus, low‐protein diet, pregnancy, rat

## Abstract

Diet intake in pregnant rats fed a low‐protein (LP) diet was significantly reduced during late pregnancy despite elevated plasma levels of ghrelin. In this study, we hypothesized that ghrelin signaling in the hypothalamus is blunted under a low‐protein diet condition and therefore, it does not stimulate diet intake during late pregnancy. Female Sprague–Dawley rats were fed a normal (CT) or LP diet from Day 1 of pregnancy. On Day 21, 0.5 *μ*g ghrelin was given into the third ventricle (ICV). Diet and water intake at 30, 60, and 120 min after ICV injection was measured. Hypothalami were dissected and analyzed for expression of genes related to appetite regulation (*Npy*,* Agrp*,* Pomc* and *Cart*) and phosphorylation of AMPK and ACC proteins (downstream proteins of ghrelin receptor activation). Results include: In response to ICV injection of ghrelin, (1) diet intake was significantly lower in LP compared to CT rats; (2) water intake was not affected in LP rats; (3) expression of *Npy* and *Agrp,* but not *Pomc* and *Cart,* were higher in the hypothalamus of LP compared to CT rats; (4) the abundance of phosphorylated AMPK and the ratio of phosphorylated to total AMPK, but not the abundance of total AMPK, were lower in LP compared to CT rats; (5) the abundance of phosphorylated ACC, but not total ACC, was lower in LP rats. These findings suggest that blunted ghrelin signaling in the hypothalamus of pregnant rats fed a LP diet leads to reduced diet intake and exacerbates gestational protein insufficiency.

## Introduction

Protein and amino acids are critical for fetal development and growth. In animals, protein insufficiency during pregnancy not only causes intrauterine growth restriction (Jansson et al. 2006; Gao et al. 2012a, 2012b), but also programs hypertension, cardiovascular and metabolic diseases in adult offspring (Langley‐Evans et al. 1996, 1999; Gangula et al. 2005; McMullen and Langley‐Evans 2005a, 2005b; Sathishkumar et al. 2012). Programming of adult health of offspring from pregnant rats fed a low‐protein (LP) diet is of clinical significance (Kautzky‐Willer and Handisurya 2009) because this model mimics protein insufficiency in the diet in developing countries, due to poverty and in certain ethnic groups in developed countries due to varied socioeconomic limitations. Recently, we found that diet consumption by rats fed a LP diet was lower during late gestation compared to that by rats fed normal protein (CT) diet (Gao et al. 2015). As a consequence, this reduced diet intake exacerbates nutritional insufficiency during late pregnancy when fetal growth is exponential. However, mechanisms for the reduced diet intake in pregnant rats fed a LP diet remain unknown.

Appetite is regulated by interaction between the central nerve system and peripheral organs. To date, ghrelin has been the only known peripherally produced hormone shown to stimulate appetite (Fernandez‐Fernandez et al. 2006; Angelidis et al. 2012), however, its orexigenic role has been questioned by *ghrl* (ghrelin) KO mice, since in these mice food intake is not reduced (Sun et al. 2003; McFarlane et al. 2014). Ghrelin is primarily produced in the stomach and released into the blood circulation. In the hypothalamus, ghrelin binds to its receptor GHSR1a (growth hormone secretagogue type 1a receptor) in NPY/AgRP (neuropeptide Y/Agouti‐related peptide) expressing neurons in the arcuate nucleus (ARC) (Kojima and Kangawa 2010), and induces phosphorylation of AMPK (AMP activated protein kinase) and ACC (acetyl‐CoA carboxylase), which promotes fatty acid metabolism and CPT1 (carnitine palmitoyltransferase) activation. Free radical production in fatty acid metabolism in the mitochondria stimulates expression of *Ucp2* (uncoupling protein 2) (Andrews et al. 2008), which is followed by increased diet intake. In addition, NPY/AgRP hormones suppress the activity of CART/POMC (cocaine–amphetamine‐regulated transcript/proopiomelanocortin) expressing neurons in the ARC (Funahashi et al. 2003; Naslund and Hellstrom 2007).

Our recent study suggested that the plasma levels of ghrelin are not associated with the changes in the diet intake in pregnant rats fed a LP diet, as elevated levels of plasma ghrelin failed to stimulate the diet intake during late pregnancy (Gao et al. 2015). This is inconsistent with the known diet intake stimulating function of ghrelin. Therefore, in this study, we hypothesized that ghrelin signaling in the hypothalamus is blunted in pregnant rats fed a LP diet and it does not stimulate diet intake during late pregnancy. We investigated the diet and water intake, expression of genes related to appetite regulation (*Npy*,* Agrp*,* Pomc* and *Cart)*, and ghrelin signaling in the hypothalamus (phosphorylation of AMPK and ACC) of pregnant rats fed a LP diet on day 21 of gestation after intracerebroventricular (ICV) injection of ghrelin.

## Materials and Methods

### Diets

The isocaloric low (6% casein)‐ and normal (20% casein)‐protein diets were purchased from Harlan Teklad (Cat. TD.90016 and TD.91352, respectively; Madison, WI). More information about these diets were described in details in our recent publication (Gao et al. 2015).

### Animals

All procedures were approved by the Animal Care and Use Committee at Baylor College of Medicine and were in accordance with those published by the US National Institutes of Health Guide for the Care and Use of Laboratory Animals (2011). Virgin female Sprague–Dawley rats weighing between 175 and 225 g, and male rats weighing between 225 and 249 g were purchased from Harlan (Houston, TX). These rats were housed in a room with a controlled light–dark cycle (light phase: 0100–1300 and dark phase: 1300–0100). Animals were allowed acclimation to our housing conditions for 1 week before cannula was implanted to the third cerebral ventricle (ICV) of female rats.

### Surgeries

Cannulas (22‐ga, 11 mm, Plastics One, Roanoke, VA) were surgically implanted into the third ventricle under isoflurane anesthesia using stereotaxic (David Kopf Instruments, Tujunga, CA) coordinates (2.2 mm posterior to bregma, 7.8 mm ventral to dura, directly on midline) as determined by the atlas of Paxinos and Watson and as previously published (Paxinos and Watson 1998; Sisley et al. 2014). Rats were allowed to recover to their presurgery body weight prior to any studies. Cannula placement was verified by a water intake of ≥5 mL, 1 h after a 5 ng/1 *μ*L injection of angiotensin II (American Peptide, Sunnyvale, CA) into the third ventricle. Only animals with a positive angiotensin response test were included in analyses.

### Animal breeding

Two weeks after ICV cannulation, 2 virgin female rats and 1 male rat were kept in a cage overnight and vaginal smear was checked under microscope. The presence of sperm in vaginal smear indicated positive pregnancy status and this day was designated as Day 1 of pregnancy. Pregnant rats were housed individually, randomly divided into two dietary groups, and received ad libitum either CT or LP diet until they were killed on Day 21 of pregnancy (*n* = 8–10 rats/diet treatment).

### Intracerebroventricular injection of ghrelin and food Intake studies

On Day 21 of pregnancy, rats were fasted for 1 h (4 h prior to dark cycle onset) in order to increase the sensitivity of detecting anorectic responses without causing compensatory hyperphagia (Ellacott et al. 2010). A quantity of 0.5 *μ*g ghrelin (1 *μ*L) was given to rats by ICV injection, 3 h prior to dark cycle onset. This dose is consistent with previously published effective doses (Nakazato et al. 2001; Lawrence et al. 2002; Clegg et al. 2003). Body weights were measured at baseline before injection. Food hoppers and water bottles were manually measured at baseline before injection, and 30, 60, and 120 min after ICV injection.

### Hypothalamic dissection, RNA extraction, reverse transcription, and real‐time PCR

The hypothalamus was collected using the dissection procedures described in a previous report (Clegg et al. 2007). Briefly, brains were rapidly removed, frozen in dry ice‐cooled isopentane, and stored at −80°C. At analyses, the hypothalamus was thawed on dry ice briefly and dissected using the tuber cinereum as the ventral landmark for cuts to remove the frontal lobe and lateral and posterior portions of the brain. The cortex was peeled away from the remaining ventral brain piece containing the hypothalamus. Total RNA was extracted from the hypothalamus by Trizol reagent (Cat. 15596‐018; Invitrogen, Carlsbad, CA) according to the manufacturer's protocol. The possible genomic DNA in total RNAs was digested with RNA‐free DNase I (Cat. 79254; Qiagen Inc., Valencia, CA), followed by cleanup procedures using a Qiagen RNeasy Minikit (Cat. 74104; Qiagen). The quality of RNA was measured by Agilent 2100 BioAnalyzer (Agilent Biotechnologies, Santa Clara, CA) and RNA integrity number of all tested RNA samples was larger than 10. cDNA was synthesized from 1 *μ*g of total RNA by reverse transcription and used for real‐time PCR with specific primers for target and housekeeping genes. Procedures and reagents for reverse transcription and real‐time PCR were described in detail in our recent report (Gao et al. 2015).

### Protein extraction and Western blotting

Hypothalamic protein was extracted from the organic phase of tissue lysates immediately after RNA extraction following standard Trizol protocol (*n* = 4/diet treatment). Procedures of protein extraction were described in detail in our previous report (Gao et al. 2012b). Aliquots of 20 *μ*g proteins were added with 4× Sample Buffer (Cat. NP0007, Invitrogen), followed by incubation at 70°C for 10 min. The separated proteins in NuPAGE 4–12% Bis‐Tris Gel (Cat. NP0321BOX, Invitrogen; for AMPK analysis) or in 3–8% Tris‐Acetate Gel (Cat. EA0375BOX, Invitrogen; for ACC analysis) were transferred onto a nitrocellulose membrane at 4°C overnight. After blocking in 5% BSA in TBST buffer, a rabbit anti‐AMPK monoclonal IgG (Cat.5832; Cell Signaling, Danvers, MA), a rabbit anti‐phosphorylated AMPK (Thr^172^) monoclonal IgG (Cat. 2535; Cell Signaling), a rabbit anti‐ACC monoclonal IgG (Cat.3676; Cell Signaling), or a rabbit anti‐phosphorylated ACC (Ser^79^) polyclonal IgG (Cat. 3661; Cell Signaling) at 1:1000 dilutions was added to nitrocellulose membrane and incubated at 4°C overnight. The blots were washed and incubated with HRP‐conjugated goat anti‐rabbit IgG (Cat. 4030‐05; Southern Biotech, Birmingham, AL) at 1:2000 dilutions at room temperature for 1 h. ACTB (*β*‐actin) was used as an internal control. Primary antibody, mouse monoclonal antibody for ACTB (Cat. 3700; Cell Signaling), and secondary antibody, HRP‐conjugated goat antimouse IgG (Cat. 1030‐05; Southern Biotech) were used at 1:10,000 dilutions. Proteins in blots were visualized with ODYSSEY FC Imaging System (LI‐COR Biotechnology, Lincon, NE) according to the manufacturer's recommendations. The relative amount of target protein was expressed as a ratio to ACTB analyzed by western blotting.

### Statistical analysis

All quantitative data were subjected to least‐squares analysis of variance (ANOVA) using the general linear models procedures of the Statistical Analysis System (Version 9.4, SAS Institute, Cary, NC). Data on diet and water intake at 30, 60, and 120 min after ICV injection of ghrelin, were expressed as the absolute weight (gram), and also as a ratio to the body weight (gram) measured before ICV injection. Data on diet and water intake, gene expression, and abundance of proteins were analyzed for the effect of diet treatment. Log transformation of variables was performed when the variance of data were not homogenous among treatment groups, as assessed by the Levene's test. A *P*‐value ≤0.05 was considered significant; a *P*‐value >0.05 and ≤0.10 was considered a trend toward significance. Data were presented as least‐squares means (LSMs) with overall standard errors (SE).

## Results

### Diet intake response to ICV injection of ghrelin was lower in pregnant rats fed LP diet

**Figure 1:**
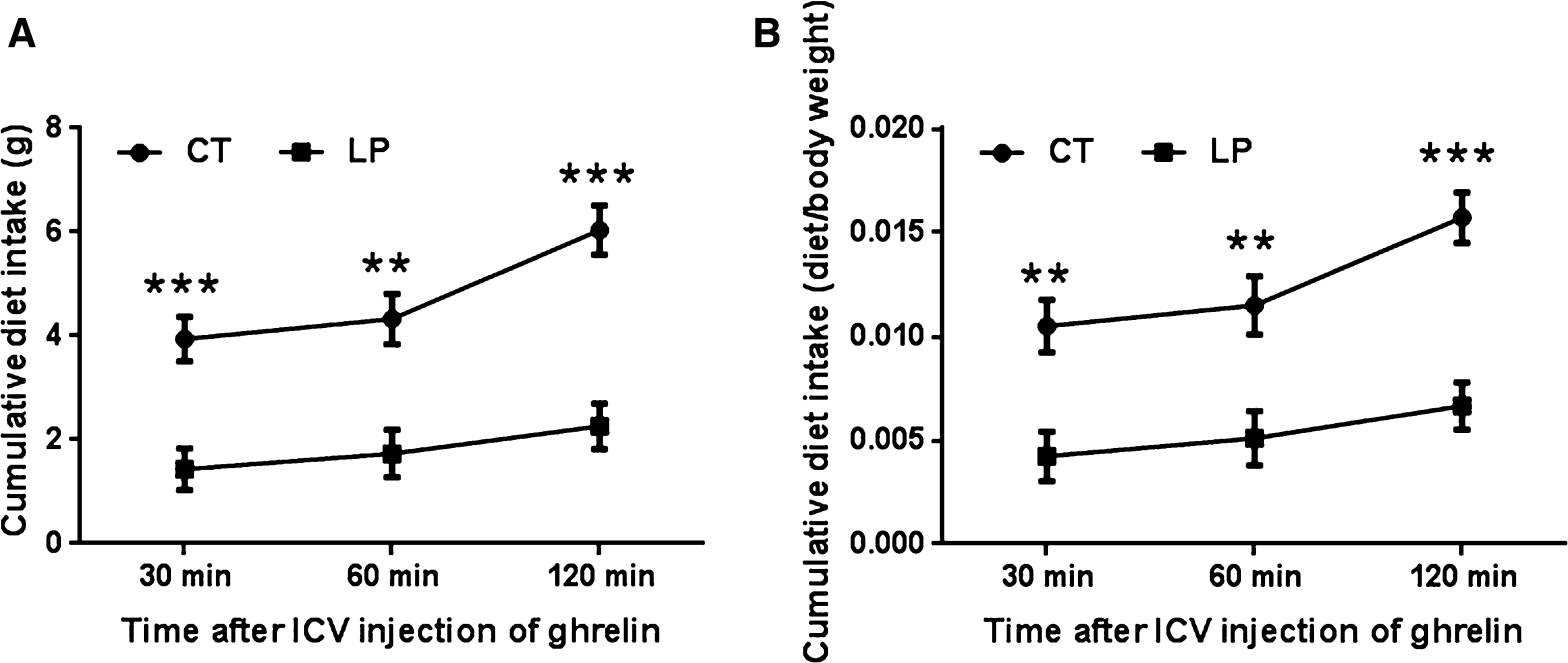
Diet intake after intracerebroventricular (ICV) injection of ghrelin. CT, control; LP, low‐protein diet. Data represents the mean ± SEM expressed as the absolute diet weight (gram) (A) or the ratio of diet intake (gram) to body weight (gram) of pregnant rats on Day 21 of pregnancy (B) (*n* = 8–10). *P <* *0.05; **P <* *0.01; ***P <* *0.001.

The cumulative diet intake expressed in absolute diet weight (g) was 2.76‐fold (*P *<* *0.001), 2.5‐fold (*P *< 0.01), and 2.67‐fold (*P *<* *0.001) higher in CT rats at 30, 60, and 120 min after ICV injection of ghrelin compared to that in LP rats, respectively (Fig. 1A). Because body weight gain in CT rats is significantly higher than that in LP rats during late pregnancy (Gao et al. 2015), diet intake was also adjusted to the weight of dams. Similarly, the cumulative diet intake expressed as the ratio to the body weight of pregnant rats (g/g) was 2.38‐fold (*P *<* *0.01), 2.24‐fold (*P *<* *0.01), and 2.38‐fold (*P *< 0.001) higher in CT rats at 30, 60, and 120 min after ICV injection of ghrelin compared to that in LP rats, respectively (Fig. 1B).

### Water intake response to ICV injection of ghrelin was not affected in pregnant rats fed LP diet

**Figure 2:**
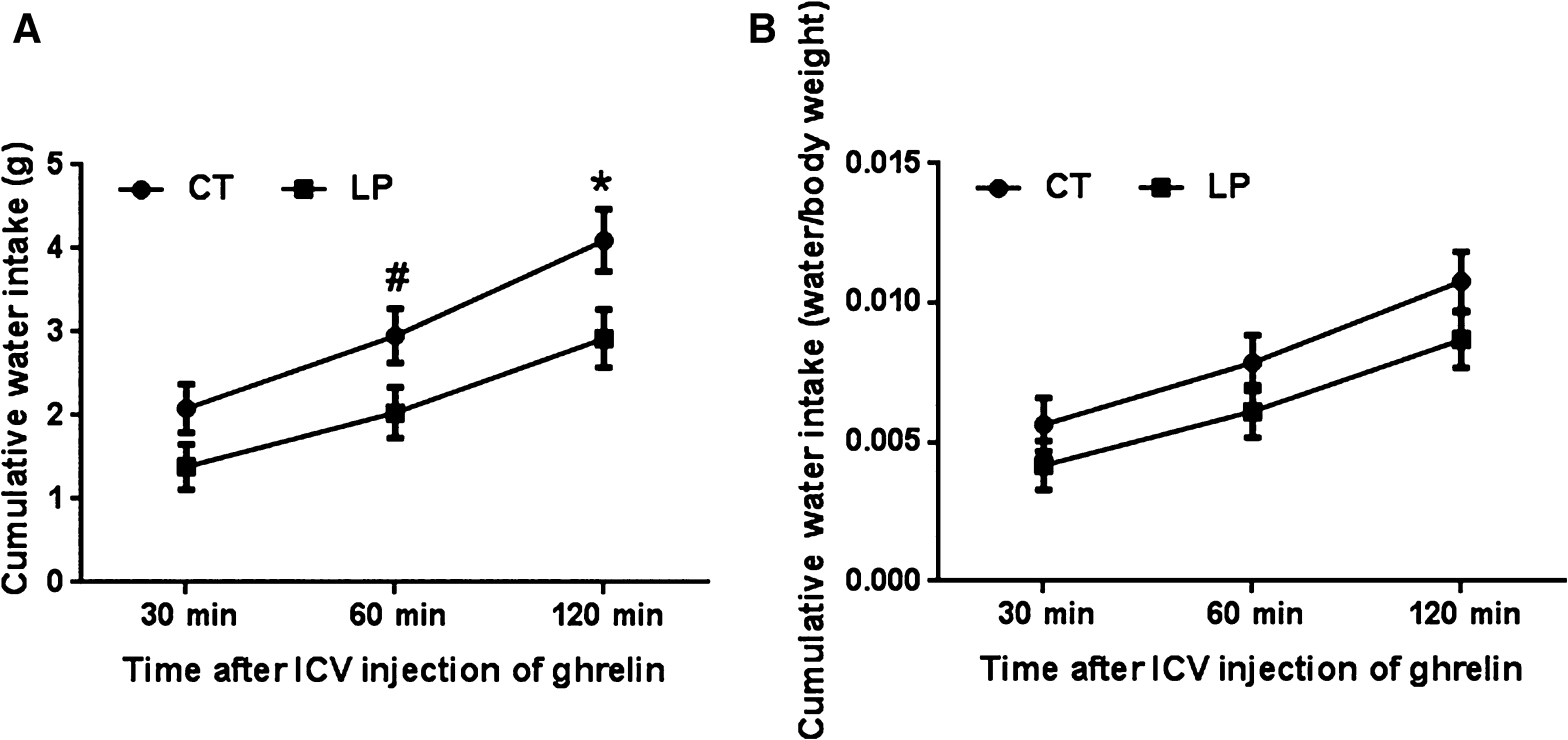
Water intake after intracerebroventricular (ICV) injection of ghrelin. CT, control; LP, low‐protein diet. Data represents the mean ± SEM expressed as the absolute water weight (gram) (A) or the ratio of water intake (gram) to body weight (gram) of pregnant rats on each day of pregnancy (B) (*n* = 8–10). *P <* *0.05; ^#^0.05 < *P *<* *0.1.

It is known that ICV injection of ghrelin can inhibit angiotensin II induced water intake (Hashimoto et al. 2010) and angiotensin II levels in pregnant rats fed the LP diet are elevated compared to the control rats (Gao et al. 2012b). To confirm the activity of ghrelin in the hypothalamus, the effect of ghrelin on water intake was investigated in this study. The cumulative water intake expressed in absolute water weight (g) was significantly higher by 1.45‐fold (0.05 < *P *< 0.1), and 1.40‐fold (*P *<* *0.05) in CT rats 60 and 120 min after ICV injection of ghrelin (respectively) compared to that in LP rats (Fig. 2A). However, the cumulative water intake expressed as the ratio to the body weight of pregnant rats (g/g) was not significantly different between groups at all the time points (Fig. 2B).

### Expression of genes related to appetite regulation in the hypothalamus in response to ICV injection of ghrelin in pregnant rats fed LP diet

**Figure 3:**
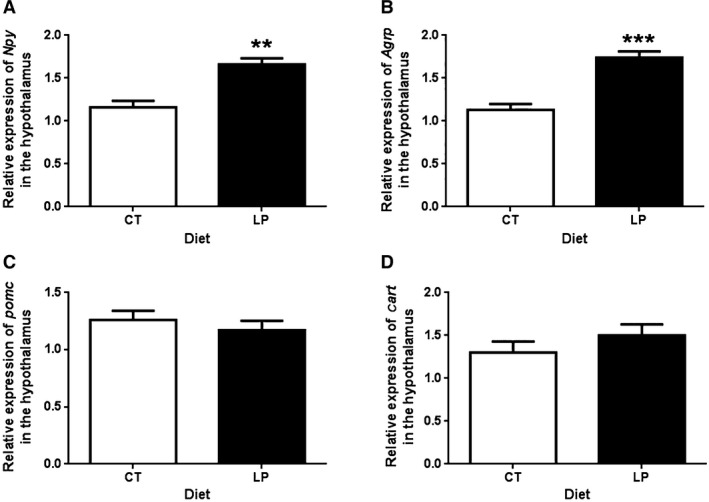
Quantitative real‐time PCR analysis of *Npy* (A), *Agrp* (B), *Pomc* (C), and *Cart* (D) in the hypothalamus after intracerebroventricular injection of ghrelin. CT, control; LP, low‐protein diet. The bar represents the mean ± SEM expressed as relative units of mRNA standardized against *Actb* (*n* = 4). **P <* *0.01; ***P <* *0.001.

Expression of genes in the hypothalamus was measured two hours after ICV injection of ghrelin. mRNA levels of appetite stimulating genes, *Npy* (Fig. 3A) and *Agrp* (Fig. 3B) were 1.67‐fold (*P *<* *0.01) and 1.74‐fold (*P *<* *0.001) higher in LP rats compared to CT rats, respectively. In contrast, mRNA levels of appetite‐inhibiting genes, *Pomc* and *Cart* (Fig. 3D) were similar between CT and LP rats.

### Phosphorylation of AMPK was reduced in the hypothalamus in response to ICV injection of ghrelin in pregnant rats fed LP diet

**Figure 4:**
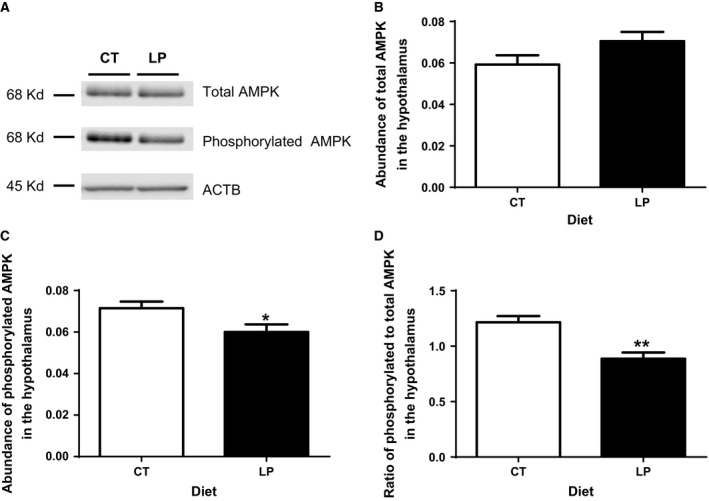
Western blotting analysis of phosphorylation of AMPK in the hypothalamus after intracerebroventricular injection of ghrelin.(A) A single band in a size of 68 kD for total and phosphorylated AMPK was detected. (B) The relative abundance of total AMPK. (C) The relative abundance of phosphorylated AMPK. (D) The ratio of abundance of phosphorylated to total AMPK. CT, control; LP, low‐protein diet. The bar represents the mean ± SEM expressed as relative abundance of protein standardized against *Actb* (*n* = 4) or the ratio of abundance of phosphorylated to total AMPK. *P <* *0.05; **P <* *0.01.

A single band was detected for total and phosphorylated AMPK proteins, respectively, by western blotting (Fig. 4A). The relative abundance of total AMPK proteins in the hypothalamus was similar between CT and LP rats (Fig. 4B). However, the relative abundance of phosphorylated AMPK protein was 1.18‐fold (*P *<* *0.05) higher in CT rats compared to LP rats (Fig. 4C). Similarly, the ratio of phosphorylated to total AMPK in the hypothalamus was 1.37‐fold (*P *<* *0.01) higher in CT rats compared to LP rats (Fig. 4D).

### Phosphorylation of ACC was reduced in the hypothalamus in response to ICV injection of ghrelin in pregnant rats fed LP diet

**Figure 5:**
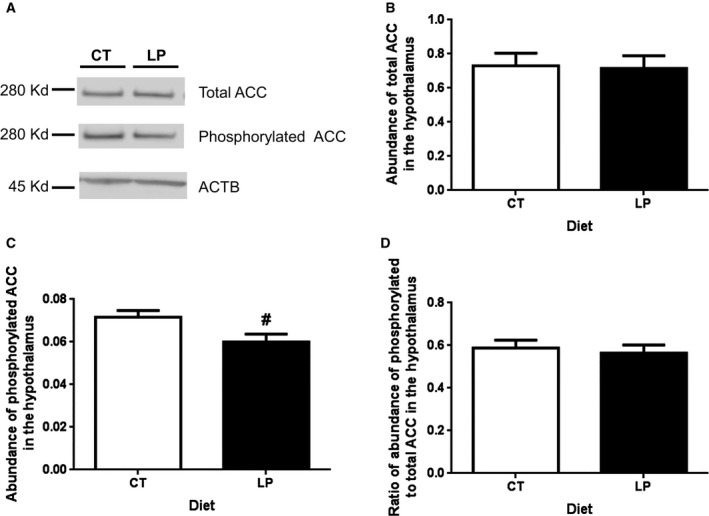
Western blotting analysis of phosphorylation of ACC in the hypothalamus after intracerebroventricular injection of ghrelin. (A) A single band in a size of 280 kD for total and phosphorylated ACC, respectively, was detected. (B) The relative abundance of total ACC. (C) The relative abundance of phosphorylated ACC. (D) The ratio of abundance of phosphorylated to total ACC. CT, control; LP, low‐protein diet. The bar represents the mean ± SEM expressed as relative abundance of protein standardized against *Actb* (*n* = 4) or the ratio of abundance of phosphorylated to total ACC. ^#^0.05 < *P *<* *0.1.

To confirm blunted ghrelin signaling in the hypothalamus of LP rats, phosphorylation of ACC, a downstream signaling protein of AMPK, was investigated. A single band was detected for total and phosphorylated ACC, respectively, by western blotting (Fig. 5A). The abundance of total ACC was similar between LP and CT groups (Fig. 5B), while phosphorylated ACC was 1.19‐fold higher (*P *=* *0.051) in CT rats compared to that in LP rats (Fig. 5C). However, the ratio of phosphorylated to total ACC was similar between the LP and CT groups (Fig. 5D).

## Discussion

Pregnancy is associated with increased diet intake, which is required to secure nutritional sufficiency for rapid fetal growth particularly during late pregnancy. Our recent study showed that pregnant rats fed a LP diet gave birth to pups that are significantly smaller compared to those that received normal protein diet (Jansson et al. 2006; Gao et al. 2012a, 2012b) and consumed a significantly lower amount of diet during later period of pregnancy (Gao et al. 2015). Offspring from mothers fed LP diet during gestation have been shown to become hypertensive when they become adults (Langley‐Evans et al. 1996, 1999; Gangula et al. 2005; McMullen and Langley‐Evans 2005a, 2005b; Sathishkumar et al. 2012). In this study, we examined if diet intake response to acute ghrelin injection into the ICV is blunted in LP diet fed pregnant rats during later period of gestation. This study for the first time demonstrated that ICV injection of ghrelin has an attenuated food intake effect in LP rats compared to CT rats. Together with our previous study (Gao et al. 2015), the current study suggests that a low‐protein diet blunts ghrelin signaling in the hypothalamus during pregnancy and therefore results in reduced diet intake during late pregnancy.

The primary objective of our current study was to investigate the role of ghrelin in pregnant rats fed the LP diet and to explore the mechanisms for fetal growth restriction in the setting of gestational protein insufficiency. Ghrelin‐induced appetite stimulation has been demonstrated in multiple publications (Kojima and Kangawa 2005; Naslund and Hellstrom 2007; Sobrino et al. 2014), although recently a few studies have reported that the genetic deletion of ghrelin did not affect appetite regulation (Sun et al. 2003; McFarlane et al. 2014). In addition to the ghrelin signaling, several other factors including endocrine, neural, and physiological adaptations also affect diet intake during pregnancy (Hirschberg 2012). In this study, the orexigenic effect of the centrally administrated ghrelin was confirmed in pregnant rats fed either the CT or LP diet. This is the first study that focuses on the role of ghrelin in diet intake during gestation, to our knowledge, while most other studies utilized male rodents. In addition, the orexigenic effect of ghrelin was potent within 30 minutes and continued for up to the examined time of 120 min after ICV injection in both CT and LP rats (Fig. 1). The diet intake within 30 and 60 after ICV injection of ghrelin as percent of total diet intake during the entire 120 min was similar in both CT and LP rats (65.2% vs. 63.3% at 30 min after ICV injection; 71.5% vs. 76.5% at 60 min in CT and LP rats), thus, the stimulatory effect of centrally administered ghrelin lasts for similar time periods between CT and LP rats. Similarly, this prolonged effect was also shown in male rats in other studies (Kojima and Kangawa 2005; Naslund and Hellstrom 2007; Sobrino et al. 2014).

The reduced diet intake response to ICV injection of ghrelin in LP rats could be explained by the lower phosphorylation of AMPK (Fig. 4), a downstream target of activated ghrelin signaling in the hypothalamus (Verhulst et al. 2012). Consequently, phosphorylation of ACC (Fig. 5), the downstream effector of AMPK trends to be lower in the hypothalamus of LP rats (Fig. 4). Although the reduction in phosphorylation of ACC was not to the same extent as that of AMPK in LP rats, this attenuated signaling from the phosphorylated AMPK to phosphorylated ACC may indicate that the impaired ghrelin signaling occurs among the upstream events of phosphorylation of AMPK phosphorylation including the Gq protein ‐coupled GHSR activation, intracellular calcium release, or autophosphorylation, and activation of Ca^2+^/Calmodulin‐dependent protein kinase, kinase 2 (Means 2008). More importantly, orexigenic effect of ghrelin depends on the metabolic status, being effective in free feeding conditions, but not effective under conditions of long‐term fasting and chronic food restriction (Alen et al. 2013). Food intake is sensitive to protein content in the diet (Journel et al. 2012), and dietary protein has greater capacity in suppressing postprandial ghrelin levels than other macronutrients (Bowen et al. 2006), and thus, a low‐protein diet increases food intake in male rats (Whitedouble dagger et al. 2000). However, it is opposite to our observations that diet intake of LP rats was lower after ICV injection of ghrelin. Moreover, our previous study shows that diet intake of LP rats in the third week of pregnancy accounts only 18.2–48.9% of that in CT rats (Gao et al. 2015), which results in both protein and caloric restriction, and may mimic a condition of chronic fasting status. If so, chronic fasting may offset partly, if not all, the orexigenic effect of ghrelin in LP rats. On the contrary, the attenuation of ghrelin's effect in LP rats may not be present in the water intake, since water intake expressed as the ratio of water weight to body weight after ICV injection was similar in the CT and LP rats (Fig. 2B). This suggests that blunted ghrelin signaling in the hypothalamus affect diet intake, but not water intake in pregnant rats fed LP diet.

In response to ICV injection of ghrelin, expression of ghrelin‐stimulated genes, *Agrp* and *Npy* in the hypothalamus in the LP rats was higher than that in CT rats (Fig. 3), but phosphorylation of AMPK, downstream target of ghrelin signaling was lower (Fig. 4). These changes are seemingly inconsistent because both gene expression of *Agrp* and *Npy* and phosphorylation of AMPK are consequences of activated ghrelin signaling and thus, they are widely used as markers for ghrelin‐induced appetite stimulation. Apparently, increased expression of *Agrp* and *Npy* could be compensatory to the reduced phosphorylation of AMPK, which could not be elucidated in this study; however, the following explanations may be helpful to understand discrepancy. First, ghrelin may independently regulate expression of *Agrp* and *Npy* and phosphorylation of AMPK and to the best of our knowledge, the direct interaction between expression of *Agrp* and *Npy* and phosphorylation of AMPK has not been reported to date. Second, factors other than ghrelin affect expression of *Agrp* and *Npy*, as *Ghsr* knockout mouse have higher expression of *Agrp* and *Npy* than wild type without administration of ghrelin (Verhulst et al. 2012). Third, ghrelin‐stimulated expression of *Agrp* and *Npy* in the hypothalamus has been reported to be dependent on glucocorticoids (Goto et al. 2006), and thus, the enhanced stimulatory effect of ghrelin on expression of *Agrp* and *Npy* in LP rats may result from elevated plasma levels of corticosterone (Zambrano et al. 2005) and/or increased expression of glucocorticoid receptor (data not shown) in the hypothalamus in LP rats after ICV injection of ghrelin. Fourth, the metabolic status of animals has direct effects on these gene expressions, but not necessarily protein phosphorylation. LP rats consumed lower amount of diet within 120 min after ICV injection of ghrelin (Fig. 1), possibly because they were still maintaining a fasting‐like status. It has been reported that mice in fasting status have higher expression of *Agrp* and *Npy*, but did not affect AMPK activity (Verhulst et al. 2012).

**Figure 6:**
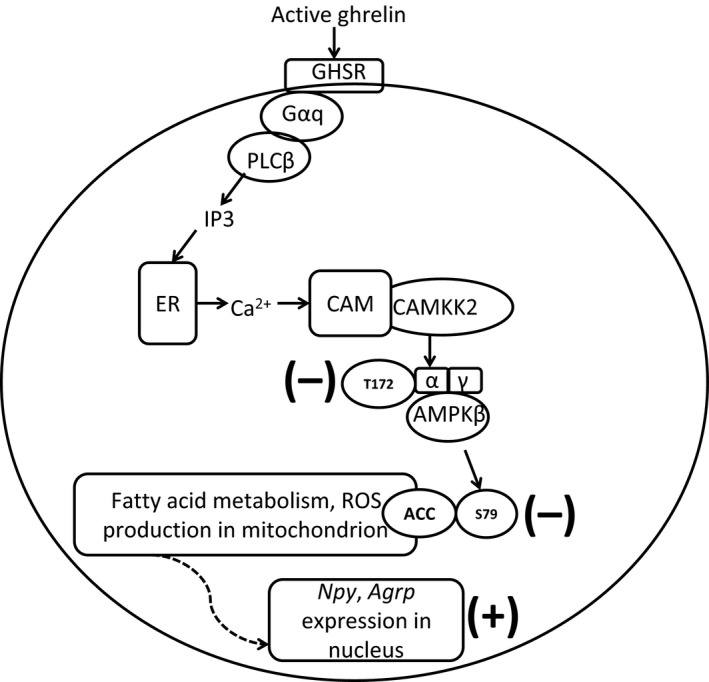
Ghrelin signaling of NPY/AgRP neurons in the hypothalamus in response to ICV injection of ghrelin in LP rats. Active ghrelin binds to its receptor GHSR and activates G*α*q and PLC *β*, which produces second messenger IP3. IP3 induces the release of calcium from ER, which activates (CAMKK2) by binding to CAM. CAMKK2 phosphorylates AMPK *α* at Threonine 172, which then phosphorylates ACC at Serine 79. Phosphorylated ACC results in enhanced fatty acid metabolism and associated production of ROS, which stimulates the expression of *Ucp2*,* Npy*, and *Agrp*, and finally, diet intake. After ICV injection of ghrelin, phosphorylation of both AMPK and ACC was lower in LP rats compared to that of CT rats (expressed in bold minus sign), but expression of *Npy* and *Agrp* was higher in LP rats (expressed in bold plus sign). GHSR, growth hormone secretagogue type 1a receptor; NPY, neuropeptide Y; AgRP, Agouti‐related peptide; G*α*q, G protein alpha q subunit; PLC *β*, phospholipase C, beta; IP3, inositol triphosphate; ER, endoplasmic reticulum; CAMKK2, calcium/calmodulin‐dependent protein kinase kinase 2; CAM, calmodulin; AMPK, AMP activated protein kinase; ACC, acetyl‐CoA carboxylase; ROS, radical oxygen species; *Ucp2*, uncoupled protein 2.

In summary, we demonstrated that the diet intake response and phosphorylation of AMPK in the hypothalamus after centrally administered ghrelin were significantly lower during later period of pregnancy in LP diet compared to CT diet fed rats. Differences between LP and CT rats in ghrelin signaling of the hypothalamus in response to ICV injection of ghrelin are schematically shown in Figure 6. As a result, ghrelin signaling in appetite stimulation is blunted in the hypothalamus of pregnant rats fed LP diet, which may explain partly, if not all, why the diet intake in pregnant rats fed LP diet was remarkably reduced in late pregnancy.

## Conflict of Interest

None declared.
